# Competency-Based Education in Low Resource Settings: Development of a Novel Surgical Training Program

**DOI:** 10.1007/s00268-017-4205-2

**Published:** 2017-09-06

**Authors:** Meghan McCullough, Alex Campbell, Armando Siu, Libby Durnwald, Shubha Kumar, William P. Magee, Jordan Swanson

**Affiliations:** 1 Division of Plastic and Reconstructive Surgery, Keck School of Medicine, University of Southern California, 1200 N. State Street, Los Angeles, CA 90033 USA; 2 Division of Plastic and Reconstructive Surgery, Children’s Hospital of Los Angeles, Los Angeles, CA USA; 3 Division of Education, Operation Smile, Virginia Beach, VA USA; 4 McGregor Comprehensive Cleft Center, Operacion Sonrisa Nicaragua, Colonia Bolonia, Managua, Nicaragua; 5 Institute for Global Health, University of Southern California, Los Angeles, CA USA; 6 Division of Plastic Surgery, Shriner’s Hospital, Los Angeles, CA USA

## Abstract

**Background:**

The unmet burden of surgical disease represents a major global health concern, and a lack of trained providers is a critical component of the inadequacy of surgical care worldwide. Competency-based training has been advanced in high-income countries, improving technical skills and decreasing training time, but it is poorly understood how this model might be applied to low- and middle-income countries. We describe the development of a competency-based program to accelerate specialty training of in-country providers in cleft surgery techniques.

**Methods:**

The program was designed and piloted among eight trainees at five international cleft lip and palate surgical mission sites in Latin America and Africa. A competency-based evaluation form, designed for the program, was utilized to grade general technical and procedure-specific competencies, and pre- and post-training scores were analyzed using a paired *t* test.

**Results:**

Trainees demonstrated improvement in average procedure-specific competency scores for both lip repairs (60.4–71.0%, *p* < 0.01) and palate (50.6–66.0%, *p* < 0.01). General technical competency scores also improved (63.6–72.0%, *p* < 0.01). Among the procedural competencies assessed, surgical markings showed the greatest improvement (19.0 and 22.8% for lip and palate, respectively), followed by nasal floor/mucosal approximation (15.0%) and hard palate dissection (17.1%).

**Conclusion:**

Surgical delivery models in LMICs are varied, and trade-offs often exist between goals of case throughput, quality and training. Pilot program results show that procedure-specific and general technical competencies can be improved over a relatively short time and demonstrate the feasibility of incorporating such a training program into surgical outreach missions.

## Introduction

Surgery is increasingly being recognized as an integral component of global health efforts. The recent *Lancet Commission* report “Global Surgery 2030” estimated that five billion people worldwide lack access to safe surgical care [1]. According to recent estimates, up to 28% of the total global burden of disease could be treated with surgery [2], but currently only 3.5% of all surgical procedures performed each year take place in low- and middle-income countries (LMICs) where the burden is highest [3]. A shortage of trained surgeons is an important contributor to this unmet need. The World Health Organization has determined that there is a “health workforce crisis” in 57 LMICs, calling current global surgical capacity “critically inadequate and grossly inequitably distributed [4].” As this crisis gains traction in global governance conversations, education is emerging as an essential component of sustainable development.

Given the overwhelming surgical need in LMICs, there exists a balance between immediate service and long-term investment in capacity building initiatives. As such, integrating educational programs into surgical aid must be implemented as effectively and efficiently as possible to maximize benefit to patients. One area of innovation in surgical education, particularly in high-income countries (HICs), has been the development of competency-based training. This approach has shown potential to reduce training time while simultaneously improving surgeon technical skill; [5, 6] however, it remains poorly understood how competency-based surgical training can be best adapted to the context of LMICs. This paper describes the development of a competency-based surgical training program, in the setting of existing surgical service initiatives, as an opportunity for accelerated specialty training of in-country providers in LMICs.

## Advances in competency-based surgical education

Traditionally, trainees have been evaluated by the global impression of supervising staff, using number of years as a benchmark of competency. However, this type of non-criterion-based rating is largely subjective and unreliable [7, 8]. Moreover, while adequate surgical volume is necessary to achieve competence, performance of a set number of procedures or completion of a certain number of years alone does not guarantee ability [9]. This realization has prompted interest in alternate educational approaches. Competency-based training is built around the evaluation of specific knowledge, skills and attitudes agreed upon as necessary for successful practice. Frequent, formative feedback provides opportunity to quickly identify deficiencies and address them in a specific and timely manner [10]. Structured evaluation also emphasizes a learner-centered approach, creating a sense of ownership and self-efficacy, which in turn contributes to the promotion of lifelong learning. Table 1 compares the time-based and competency-based approaches.

**Table 1:** Comparison of time-based versus competency-based educational approaches (adapted from Carraccio et al. [10])

	Time-based	Competency-based
Driving force of curriculum	Content-knowledge acquisition	Outcome-knowledge application
Driving force for process	Teacher	Learner
Setting of evaluation	Removed (gestalt)	Direct observation
Basis of evaluation	Norm-based	Criterion-based
Program completion	Fixed time	Variable time

Over the last decade, HICs have increasingly moved toward this competency-based model in medical education. Initiatives through the Accreditation Council for Graduate Medical.

Education (ACGME) in the USA, the Royal College of Physicians and Surgeons of Canada and a number of European and Australian counterparts have sought to increase documented competency as a way of improving public accountability [11, 12]. Most training programs have maintained a hybrid of time and competency-based approaches, but several have trialed purely competency-based models with promising results [5, 6, 13]. The University of Toronto pilot program in orthopedic surgery found sustained improvement in resident technical ability, and comparable, if not higher, in-training examination scores [6]. Residents in the experimental cohort also completed the residency program a full year earlier, on average, than the residents in traditional model [6].

## The case for competency-based education in LMICs

The educational theory of deliberate practice describes the essential components of acquisition of technical skills as intense, repetitive performance of an intended skill coupled with rigorous assessment [14, 15]. However, such consistent repetition may not always be feasible in clinical practice, especially for less common conditions such as cleft lip and palate. In collaboration with the University of Southern California, Operation Smile saw a unique opportunity to train in-country providers given the high case volumes in LMICs and the immediate repetition of specific procedures inherent to Operation Smile’s service model. When coupled with frequent, targeted feedback, this environment holds even further potential to expedite the learning curve [16]. The University of Southern California and Operation Smile therefore created a “fellowship without borders,” combining intensive, focused training on short-term service initiatives with competency-based evaluation to accelerate the training of in-country surgeons in cleft-specialty techniques.

## Pilot study methods

The program was piloted at five sites (Nicaragua, Paraguay, Mexico, Guatemala and Malawi) between November 2016 and April 2017. At each site between one and three, local trainees were identified in collaboration with existing in-country senior surgeons and local medical foundations. Each individual had completed plastic or maxillofacial training in their respective country, but with minimal to no experience in cleft lip and palate surgery. These trainees were selected for their expressed commitment to deliver cleft surgical care within their countries and their ability to participate in the entirety of the training program. Prior to the mission, they were provided with a comprehensive cleft surgery manual, designed specifically for the program, as well as access to an online repository of surgical educational materials. During the week-long mission, they were formally paired with two different mentor surgeons on alternate days and worked on a designated operating room table.

A core component of the program was the development of a competency-based evaluation instrument, which assessed a combination of general technical and procedure-specific skills. The general technical skill section was adapted directly from the objective structured assessment of technical skills (OSATS), a validated instrument in multiple specialties of surgery [17]. The procedure-specific skill section was derived through a Delphi method to determine the essential steps of unilateral lip, bilateral lip and palate procedures from existing Operation Smile senior surgeons. While to the authors’ knowledge there are no current instruments for cleft surgery, similar validated procedure-specific instruments in other specialties exist. The gastrointestinal endoscopy competency assessment tool (GiECAT) [18] and the global operative assessment of laparoscopic skills (GOALS) [19] similarly define essential components of selected procedures and were used as models. A category for pre- and postoperative care was also included and was similarly modeled off the GiECAT. All sections were graded on a five-point Likert scale reflecting the degree of supervision the trainee required to perform the task, and a free text space was provided in each category for additional comments. The evaluation form is attached for reference as supplemental material.

The evaluation was completed by the mentor surgeon after the first day to characterize baseline ability, and again after the last day of the program to track incremental improvement. Raw scores were converted into percentages based on the total possible score for each section, and pre- and post-training scores were analyzed using a paired *t* test with SPSS statistical software. Within procedure-specific criteria, individual technical components were analyzed for percentage improvement in unilateral lip and palate procedures, but there was insufficient data to analyze bilateral lip procedures.

## Results

**Fig. 1:**
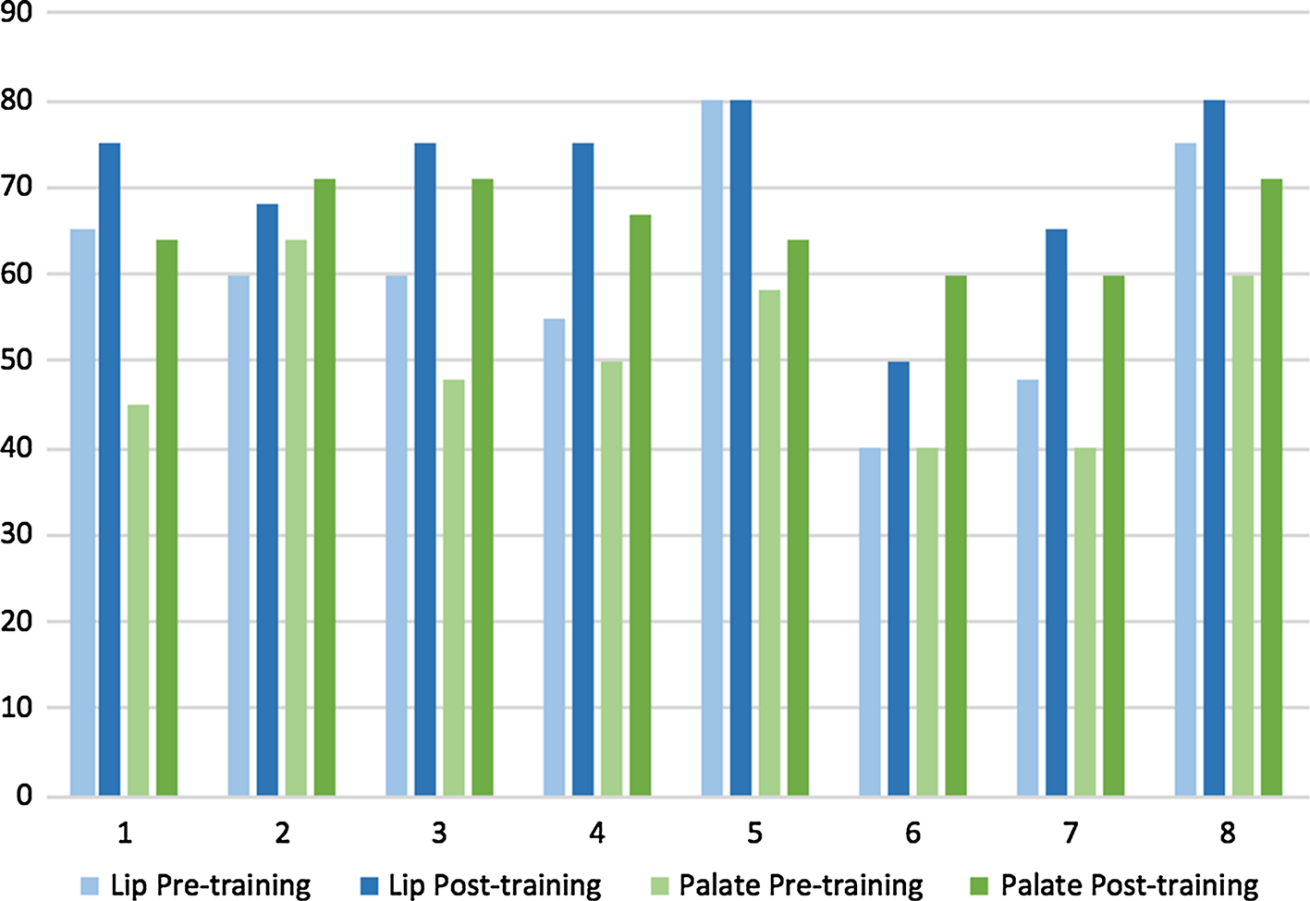
Percent score by trainee for unilateral lip- and palate-specific competencies, pre- and post-training

**Fig. 2:**
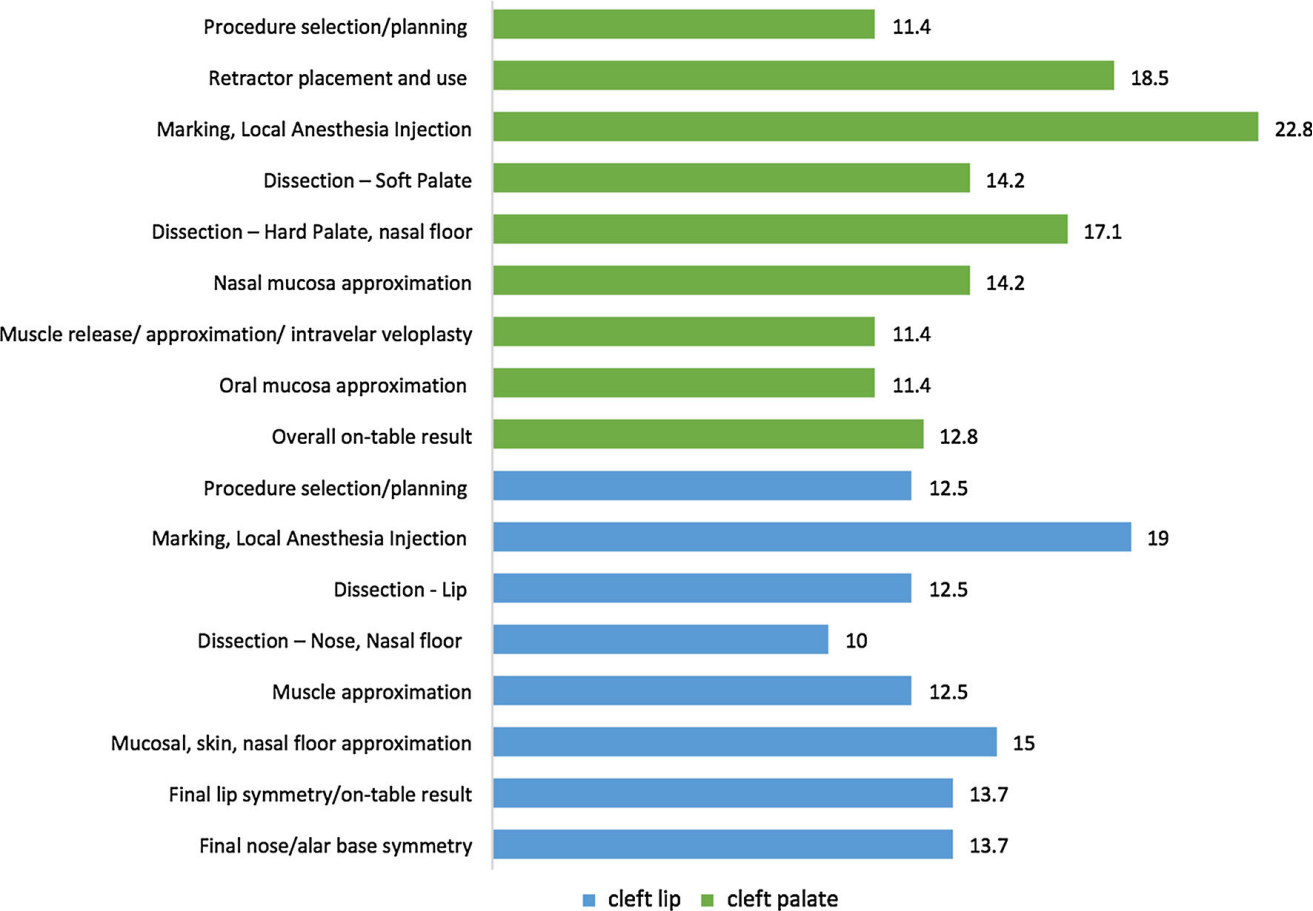
Average percentage improvement by procedure component for unilateral lip and palate

A total of eight trainees participated in the program over the five pilot sites with all candidates demonstrating improvement in each of the evaluated competencies. Average general technical competency scores improved from 63.3 to 72.0% (*p* < 0.01) and as did procedure-specific competency scores for both cleft lip repairs, from 60.4 to 71.0% (*p* < 0.01), and cleft palate repairs, from 50.6 to 66.0% (*p* < 0.01). Postoperative care competencies improved from 64.8 to 72.8% (*p* < 0.05), while preoperative care competencies improved from 66.2 to 75.0%, trending toward significance (*p* = 0.06). Average percent scores, percentage improvement and *p* values for preoperative, postoperative, general technical and procedure-specific competencies are listed in Table 2. The greatest average percentage improvement was found in palate-specific competencies (30.4%), followed by lip-specific competencies (17.5%). Figure 1 demonstrates the percent score by individual trainee for unilateral lip and palate procedures pre- and post-training. The majority of trainees started with a higher baseline percent score in unilateral lip procedures, but showed a greater percentage improvement in palate procedures. Among the procedural competencies assessed within unilateral lip, surgical markings showed the greatest improvement (19.0%) followed by mucosal/skin/nasal floor approximation (15.0%). For the palate, markings again showed the greatest improvement (22.8%), followed by retractor placement (18.5%) and quality of dissection of the hard palate/nasal floor (17.1%). Percentage improvement for each component of the procedures is detailed in Fig. 2.

**Table 2:** Average percent score (with standard deviation), percentage improvement and *p* value (with 95% confidence interval) for evaluated competencies

Competency	Pre-training %	Post-training %	% Improvement	*p* value (95% CI)
Preoperative	66.2 (±7.44)	75.0 (±9.25)	13.2	0.06 (−0.6, 18.1)
Postoperative	64.8 (±12.63)	72.8 (±14.36)	14.4	<0.05 (1.8, 16.6)
General	63.6 (±10.05)	72.0 (±6.41)	13.2	<0.01 (3.7, 12.9)
Lip-specific	60.4 (±13.19)	71.0 (±9.97)	17.5	<0.01 (5.1, 16.1)
Palate-specific	50.6 (±9.14)	66.0 (±4.72)	30.4	<0.01 (9.9, 20.7)

## Discussion

There has been a call in the literature to link mission-based work with surgical training [20], but to our knowledge, this is the first proposed program that utilizes competency-based principles to maximize the unique training opportunities presented by the surgical mission setting. The “fellowship without borders” utilizes existing models of care delivery to train in-country providers and improve education and sustainability while simultaneously continuing to provide much needed services. It shows potential to expedite the learning curve in cleft surgical training by capitalizing on the high case volume and procedure repetition inherent to the mission setting. The competency-based approach maximizes efficiency through objective, structured evaluation and assesses valuable data lacking in many other educational initiatives to monitor skill acquisition and maintenance and adapt appropriately to changing needs.

It is estimated that participation with the program over three- to four-week-long experiences will be required to reach competency, based on the trainee’s baseline evaluation level. At this time, none of the trainees have completed scheduled follow-up training in order to assess skill retention between training sessions or to confirm the estimated trajectory to competency, and further data is expected to refine programmatic design. During the program, a case log is completed by the trainee each day, and this will be used in future analysis to draw associations between the number of cases necessary, on average, to reach an objectively evaluated level competency in different procedures. One of the advantages to competency-based evaluation is that allows for variable timing of skill progression, and it is expected that trainees will move through the program at different rates. Nonetheless, correlations with average case numbers may be helpful in setting expectations for trainees and mentors, aiding in organizational oversight and logistical planning and adapting the program to fit trainee needs. Finally, as the program progresses, information on case numbers outside of the formal education setting provided during the mission will be examined to demonstrate the impact of training on patients’ access to care.

While the number of participants is limited and the results are early, the program shows promise in improving the skills of in-country surgeons in cleft-specialty procedures in an evidence-based fashion. Several characteristics of the program contribute to its potential. An emphasis on frequent, structured assessment of trainee skill progression allows for adaptation of the program to trainee needs as well as systematic program evaluation. While the intent of the program is the development of competency in cleft procedures in the trainees’ own local practice, additional summative evaluation with respect to Operation Smile credentialing encourages trainees to maintain connection to the organization. This facilitates long-term follow-up and closer monitoring of skill maintenance over time, which is essential to determining the program’s success and/or identifying additional training needs. Support from local surgical staff is also critical to the success of the program. The pilot utilized both US-based surgeons and Nicaraguan, Malawian, Guatemalan, Paraguayan and Mexican surgeons as its senior mentors, fostering investment from the local surgical community. Future steps include collaboration with national credentialing bodies to allow credentialing from the program to be recognized locally. This process is necessarily country-specific and will require additional data before formal agreements can be reached.

Although promising, several challenges face the program. The length of the evaluation form was cited by mentors as a limitation. It is currently two pages but was still be felt to be time consuming, especially when required multiple times over the course of the mission. Future refinement will need to balance collection of robust data with minimization of paperwork. Additionally, the program is dependent on the commitment of both surgical mentors and trainees to engage in multiple training sessions over the course of several missions. Given the time away from existing practice responsibilities required to attend missions, consistent participation could be difficult, making appropriate participant selection essential. Finally, although the assessment tools are comprehensive, they are completed by the mentor surgeons and confounding factors such as observer bias cannot be excluded.

## Conclusion

As surgical disease is being brought into the global health spotlight, there is increasing recognition that education is key to addressing the vast burden of disease. Designing and evaluating educational initiatives in a complex field such as surgery is highly challenging, and the complexities of training only become further amplified in the global arena. Multiple educational initiatives have been previously implemented, each with their own strengths and limitations, but consensus is growing that these must continue to improve to keep pace with the increasing number of patients and the challenges of delivering care. To meet this challenge, Operation Smile and the University of Southern California propose a “fellowship without borders,” built on the principles of competency-based education and utilizing existing service platforms. This program leverages high patient volumes in the mission setting to more rapidly train in-country providers and gathers valuable data to systematically evaluate and improve the program. Further data collection is ongoing to determine the validity of the evaluation instrument, the generalizability of pilot results and the impact of training on surgical disease burden.
